# Advances in the optimization of central carbon metabolism in metabolic engineering

**DOI:** 10.1186/s12934-023-02090-6

**Published:** 2023-04-21

**Authors:** Zhenke Wu, Xiqin Liang, Mingkai Li, Mengyu Ma, Qiusheng Zheng, Defang Li, Tianyue An, Guoli Wang

**Affiliations:** 1 Featured Laboratory for Biosynthesis and Target Discovery of Active Components of Traditional Chinese Medicine, School of Integrated Traditional Chinese and Western Medicine, Binzhou Medical University, Yantai, 264003 China; 2 Yantai Key Laboratory of Pharmacology of Traditional Chinese Medicine in Tumor Metabolism, School of Integrated Traditional Chinese and Western Medicine, Binzhou Medical University, Yantai, 264003 China

**Keywords:** Central carbon metabolism, Metabolic engineering, Microbial chassis, Metabolic pathways, Yeast, *Escherichia coli*

## Abstract

Central carbon metabolism (CCM), including glycolysis, tricarboxylic acid cycle and the pentose phosphate pathway, is the most fundamental metabolic process in the activities of living organisms that maintains normal cellular growth. CCM has been widely used in microbial metabolic engineering in recent years due to its unique regulatory role in cellular metabolism. Using yeast and *Escherichia coli* as the representative organisms, we summarized the metabolic engineering strategies on the optimization of CCM in eukaryotic and prokaryotic microbial chassis, such as the introduction of heterologous CCM metabolic pathways and the optimization of key enzymes or regulatory factors, to lay the groundwork for the future use of CCM optimization in metabolic engineering. Furthermore, the bottlenecks in the application of CCM optimization in metabolic engineering and future application prospects are summarized.

## Background

Since its inception, metabolic engineering has rapidly promoted the biosynthesis of natural products [1, 2]. In the pharmaceutical industry, various high-value pharmaceutical ingredients, including artemisinin, ginsenosides, opioids and vinblastine, have been synthesized using microorganisms [3–6]. The growing environmental awareness of the general public and the scarcity of fossil fuels have gradually revealed the advantages of green biomanufacturing of bulk chemicals [7, 8]. The rise in global oil prices has also heightened interest in biofuels [9, 10].

Metabolic engineering employs genetic engineering techniques to increase the yield of target products by modifying metabolic pathways within cells. The most common methods in metabolic engineering include manipulation of promoter and copy number of target enzymes [11, 12], transcription factor regulation [13], fusion protein construction [14], protein scaffold assembly [15], organelle compartmentalization [16], and dynamic regulatory engineering [17]. The integration of these approaches enables complex and sophisticated metabolic pathway optimization of chassis cells to develop desired optimization strategies such as increasing the metabolic flux of target product-related pathways [18], blocking or attenuating other target product-consuming pathways [19], increasing the catalytic rate of rate-limiting steps [20], and introducing heterologous metabolic pathways [21]. However, a wide range of engineering modifications can have varying degrees of negative impact on the overall metabolism of the chassis strain, causing an imbalance in the metabolic flux of the chassis strain, inhibiting its physiological activity, and ultimately affecting production performance [22]. Because the optimization of the metabolic pathways where the target products are located or adjacent has been relatively well established, further optimization of these pathways has a limited effect on yield improvement. Therefore, recent studies have focused on the global regulation of metabolic flux, looking for breakthroughs in the most fundamental metabolic pathway, central carbon metabolism (CCM), which includes glycolysis, the tricarboxylic acid cycle (TCA cycle), and the pentose phosphate pathway (PPP).

CCM is a major source of energy for cell growth and development and provides precursors for other metabolic activities. Modification of the CCM, which is upstream of biological metabolic activities, often results in the rearrangement of the global metabolic flux of the cell and has a high potential for metabolic engineering applications. On the one hand, optimization of CCM can increase the precursors supply for the targeted compounds. For example, the introduction of the heterologous phosphoketolase phosphotransacetylase (PHK) metabolic pathway can increase the rate of acetyl coenzyme A (acetyl-CoA) synthesis and trigger CCM (glycolysis and the PPP) rearrangement in *Saccharomyces cerevisiae*, thereby increasing the production of the target product farnesene by 25% [23]. On the other, the manipulation of CCM often causes the rebalance of the availability of energy and the redox cofactors, such as ATP (adenosine triphosphate), NADPH (nicotinamide adenine dinucleotide phosphate) and NADH (nicotinamide adenine dinucleotide), to promote the output of final products by improving the corresponding catalytic steps involved in the biosynthesis pathways. The introduction of the *Deinococcus radiodurans* response regulator DR1558 into *E. coli* improves the expression efficiency of the genes related to CCM, and induces the excess generation of NADPH from PPP and supplies the cofactor requirements during PHB biosynthesis [24].

In recent years, metabolic engineering strategies on the optimization of CCM has produced remarkable results in the biosynthesis of many natural products. However, the current status of the application of this optimization strategy has not yet been systematically discussed. In this work, representative chassis strains of yeast and *E. coli* were selected to summarize the application and potential of CCM in metabolic engineering.

## Application of CCM optimization in yeast

### Introduction of heterologous metabolic pathways

**Fig. 1:**
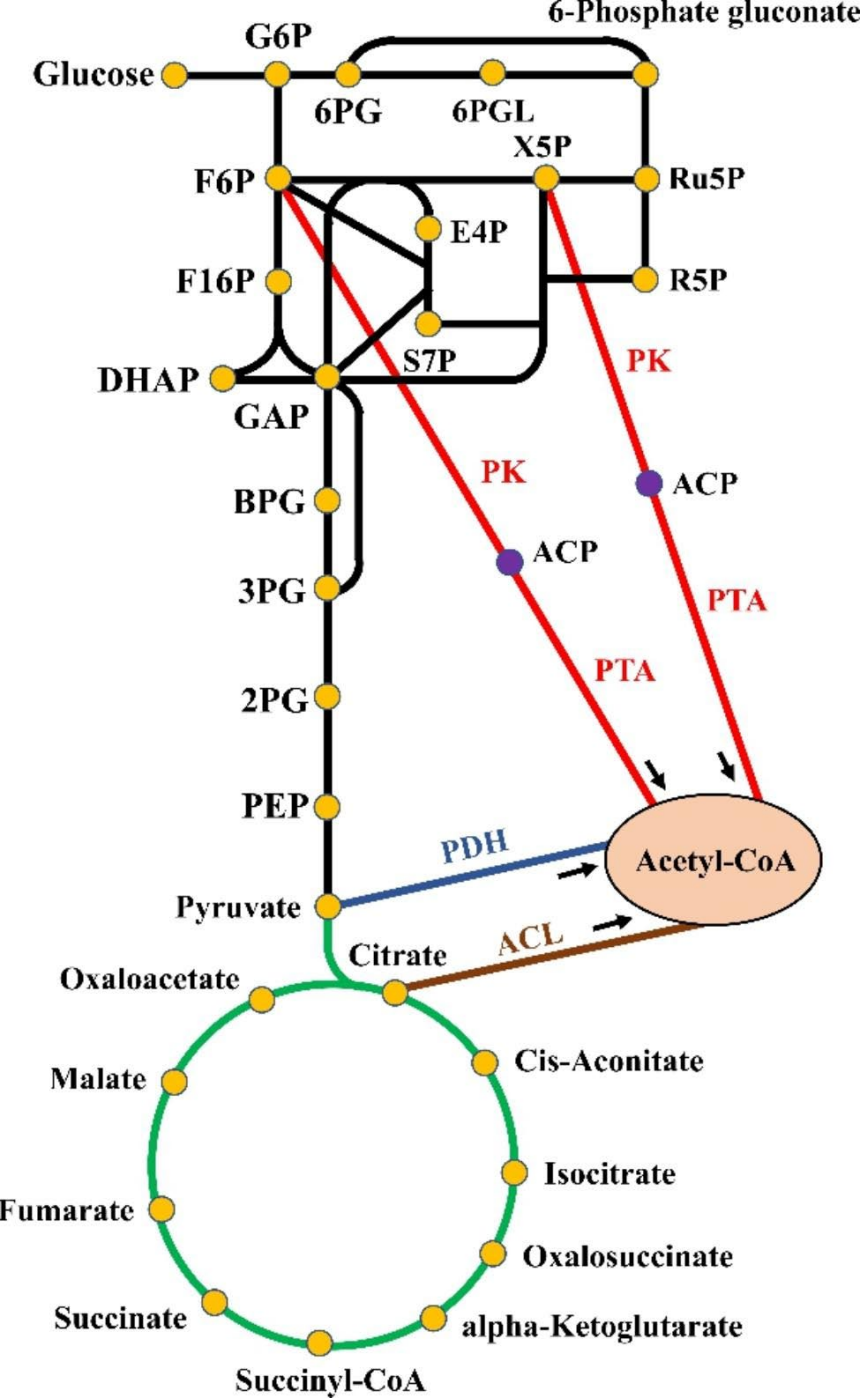
The optimization of CCM by introduction of heterologous pathways to improve the carbon flux in *S. cerevisiae*. The black line represented glycolysis and pentose phosphate pathway, and the green line represented the tricarboxylic acid cycle. The red, blue and brown lines represented the implemented PHK, PDH and ACL pathways. The orange dot represented the products in CCM, while the purple dot represented ACP. G6P, Glucose 6-phosphate; F6P, Fructose-6-phosphate; F1,6P, Fructose-1,6-bisphosphate; GAP, Glyceraldehyde 3-phosphate; DHAP, Dihydroxyacetone phosphate; BPG, 1,3-Bisphosphoglycerate; 3PG, 3-Phosphoglycerate; 2PG, 2-Phosphoglycerate; PEP, Phosphoenolpyruvate; 6PG, 6-Phosphogluconate; 6PGL, 6-Phosphate glucono-1,5-lactone; X5P, Xylulose 5-phophate; Ru5P, Ribulose 5-phophate; R5P, Ribose 5-phosphate; E4P, Erythrose 4-phosphate; S7P, Sedoheptulose 7-phophate; ACP, Acetyl-phosphate; Acetyl-CoA, Acetyl coenzyme A; PK, Phosphoketolase; PTA, Phosphotransacetylase; PDH, Pyruvate dehydrogenase; ACL, ATP: citrate lyase

The introduction of a heterologous CCM metabolic pathway has been shown to be an effective method for regulating CCM in host cells. The introduction of a heterologous CCM that is not found in *Saccharomyces* species could improve the carbon flux between different pathways of CCM, and promote the biosynthesis of target compounds (Fig. 1).

#### PHK pathway

In the CCM of yeast, glucose-6-phosphate (G6P) can generate either fructose-6-phosphate (F6P) in glycolysis or ribulose-5-phosphate (Ru5P) and xylulose 5-phophate (X5P) into the PPP pathway. The introduction of PHK in *S. cerevisiae* catalyzes the direct production of acetyl-phosphate (ACP) from F6P and X5P to acetyl-CoA via a transacetylation reaction [25]. The only enzymes in the PHK pathway are phosphoketolase (PK) and phosphotransacetylase (PTA). Due to its simple constitution, it is widely used in metabolic engineering.

The PHK pathway facilitates the direct synthesis of acetyl-CoA and the biosynthesis of lipid compounds using acetyl-CoA as a precursor. The knockout of phosphofructokinase (PFK) in *Yarrowia lipolytica* blocked the metabolic flux of G6P in glycolysis and caused the redox imbalance with excess NADPH production. Then the introduction of the PHK pathway resulted in the accumulation of acetyl-CoA and correction of the redox imbalance by providing a route towards the NADPH-oxidizing lipid synthesis pathway, and a 19% increase in total lipid production was derived in the host strain [26]. In the TCA cycle, citrate can be catalyzed by ATP:citrate lyase (ACL) to produce acetyl-CoA [27]. The mouse-derived ACL and PHK pathway were used to optimize the CCM in fatty acid biosynthesis in *Pichia pastoris*. Coupled with the subsequent overexpression of NADPH-generating enzymes in TCA cycle and PPP, the engineered strain produced 23.4 g/L of free fatty acids and 2.0 g/L of fatty alcohols [28]. Overexpression of alcohol dehydrogenase 2 (Adh2), acetaldehyde dehydrogenase 6 (Ald6), and exogenous acetyl-CoA synthetase (ACS) variant acs_SE_^L641P^ following the introduction of the PHK pathway, which provides acetyl-CoA and NADPH, in *S. cerevisiae* inhibited the synthesis of ethanol and yielded 5100 ± 509 g/CDW (cell dry weight) of fatty acid ethyl esters [29].

The introduction of the PHK pathway also addresses the issue of insufficient erythrose-4-phosphate (E4P) synthesis in *S. cerevisiae*. The PHK pathway catalyzes the conversion of F6P to acetyl-CoA, which decreases the consumption of metabolic flux in glycolysis while indirectly increasing the metabolic flux in the PPP and promoting E4P accumulation, which provides a large number of precursors for the synthesis of aromatic compounds. The introduction of the PHK pathway in *S. cerevisiae* shifts the glycolytic flux to E4P synthesis, avoiding the loss of metabolic flux at multiple steps upstream of the glycolysis and PPP. The subsequent promoter optimization and dynamic regulation resulted in a yield of 12.5 g/L for p-hydroxycinnamic acid and a maximum yield on glucose of 154.9 mg/g [30]. The heterologous PHK pathway can increase tyrosol production in the host strain by 135-fold by rearranging the glycolysis and the PPP. Fed batch fermentation using glucose as a carbon source resulted in a total tyrosol and salidroside production of over 10 g/L [31]. Therefore, exogenous introduction of the PHK pathway is an effective strategy for the synthesis of aromatic compounds and derivatives downstream of the PPP pathway, especially in the absence of the available precursor E4P. However, the introduction of the PHK pathway had no significant effect on the biosynthesis of other aromatic compounds. The expression of the PHK pathway did not significantly increase the yield of 2-phenylethanol (2-PE), which may be attributed to the excess carbon flux from pyruvate synthesis that the PHK pathway competes for [32]. Hence, when multiple CCM pathways are regulated in parallel, the introduction of the PHK pathway may be subjected to unknown interference.

The regulatory strategy of the PHK pathway in chassis strain has also been applied to the biosynthesis of other classes of compounds. Protopanaxadiol (PPD), an active triterpene compound, serves as a precursor of high-value ginsenosides. The introduction of the PHK pathway and multicopy integration of endogenous transaldolase 1 (Tal1) and transketolase 1 (Tkl1) in *S. cerevisiae* increased the PPD yield to 152.37 mg/L [33]. In addition, the introduction of the PHK pathway in *S. cerevisiae* increased 3-hydroxypropionic acid (3-HP) production by 41.9% and decreased glycerol production by 48.1%. In addition, reducing the expression of phosphoglucose isomerase and overexpressing acetyl-CoA carboxylase (Acc1) and malonyl-CoA reductase (MCR) promoted the metabolic flux to the PPP, yielding 864.5 mg/L of 3-HP (24 times the yield of the initial strain) [34]. Furthermore, after being introduced into *S. cerevisiae* strain containing the ethanol degradation pathway, the *Aspergillus nidulans*-derived PHK pathway could be used for the synthesis of polyhydroxybutyrate (PHB) with a yield of 56.4 mg/g in ethanol medium [25].

#### Other heterologous pathways

Other pathways, in addition to the PHK pathway, can modulate the CCM of the yeast chassis. ACL from *A. nidulans* increased the mevalonate yield to 2-fold by directly converting citric acid to acetyl-CoA in the TCA cycle of *S. cerevisiae* [35]. But this strategy is unsuitable for large-scale industrial production due to the limitation of the citric acid flux of the ACL substrate. Besides, the pyruvate dehydrogenase (PDH) pathway of *E. coli* can directly convert pyruvate from the glycolytic pathway to acetyl-CoA. The absence of ATP consumption in this process conserves more energy for other CCM reactions. After NADP^+^-dependent modifications in *S. cerevisiae*, the introduction of PDH pathway resulted in a 2-fold increase in acetyl-CoA [36]. Despite the inferiority of the regulatory effect of the above pathways on CCM compared to the PHK pathway, there may be synergistic effects between these pathways and the PHK pathway. A-ALD in *E. coli* can not only catalyze the conversion of acetaldehyde to acetyl-CoA, but also promotes the accumulation of large amounts of redox cofactor NADH in the cytoplasm, which would balance the relationship between NADPH consumption and NADH generation in yeast cells [37, 38]. Then the combined use of the A-ALD and PHK pathways in *S. cerevisiae* resulted in the accumulation of large amounts of acetyl-CoA. Optimization on this basis yielded 279.0 ± 13.0 mg/L of β-amyrin [38].

### Optimization of CCM by key enzymes or regulatory factors in yeast

#### Optimization of key enzymes

The modification of key enzymes in each CCM pathway can rearrange the metabolic flux and facilitate the synthesis of target products. Pyruvate decarboxylase (PDC) initiates the catalysis of pyruvate (a product of glycolysis) to ethanol in *S. cerevisiae*. The knockdown of the PDC gene effectively shifts the metabolic flux from the ethanol synthesis pathway to the pyruvate-related pathway in the CCM, reducing consumption of the CCM flux by the ethanol synthesis pathway. Pyruvate accumulation was also confirmed by deletion mutants of *pdc1* and *pdc5* in *S. cerevisiae* [39]. A yield of 81.0 g/L of 2,3-butanediol was attained by reconstituting the 2,3-butanediol biosynthetic pathway in PDC knockout strains and performing batch fermentation with high concentrations of glucose as a carbon source [40]. In *S. cerevisiae*, knocking out hexokinase 2 (*HXK2*) and glucokinase 1 (*GLK1*) in glycolysis and using tetracycline transactivator protein (tTA) to control hexokinase 1 (*HXK1*) transcription can shift the metabolic flux from glycolysis to the gluconate synthesis pathway, promoting efficient gluconate biosynthesis, with the final strain showing a 50-fold increase in gluconate production compared to the control strain [41]. Overexpression of glucose-6-phosphate dehydrogenase (Zwf), glucose-6-phosphate isomerase (Pgi), and Pfk1 in *P. pastoris*, which effectively inhibits the carbon flux of glycolysis, can promote inositol biosynthesis, with inositol production reaching 30.71 g/L [42].

The above reports were optimized for only a few (1–3) key enzymes in CCM. In the metabolic engineering of some target compounds, large-scale optimization of enzymes in CCM has also been performed. The knockdown of 15 relevant CCM enzyme genes in *S. cerevisiae* and the introduction of 2-pyrone synthase (2-PS) in *Gerbera hybrida* promote the decarboxylation/condensation reaction of acetyl-CoA and malonyl coenzyme A (malonyl-CoA) to produce triacetic acid lactone (TAL). This process ultimately leads to a 37-fold increase in TAL yield to 2.2 g/L and a 50-fold increase on glucose yield to 0.13 g/g [43].

#### Optimization of key regulatory factors

In yeast, acetyl-CoA is primarily derived from CCM. The partitioned distribution and insufficient amount of acetyl-CoA limit the ability to synthesize the target product. In Crabtree-negative strain *Komagataella phaffii*, an ethanol-inducible and constitutive transcriptional regulatory signaling amplifier designed with the transcription activation region of the transcription factor MIT1 can increase ethanol-inducible expression capacity by nearly 20-fold. In addition, this process enables host cells to produce acetyl-CoA independently of CCM using ethanol as a fermentation substrate as well as an acetyl-CoA precursor and inducer. Direct production of acetyl-CoA from ethanol in the cytoplasm via a three-step catalytic process and construction of a biosynthetic pathway increased the yield of the cholesterol-lowering drug simvastatin intermediate, monacolin J, to 3.2 g/L [44].

Ric1 is a transcriptional repressor of multiple genes in the aromatic amino acid biosynthetic pathway in *S. cerevisiae* [45]. Decreasing Ric1 expression in *S. cerevisiae* and overexpressing ribose-5-phosphate ketol-isomerase (Rki1) in the PPP resulted in a 7-fold increase in the yield of shikimic acid (SA) to 2.5 g/L. The total yield of muconic acid and the intermediate product protocatechuic acid in the strain developed on this basis was 2.7 g/L [46]. A summary of the applications of CCM optimization in yeast were provided in Table 1.

**Table 1:** Optimization of CCM in eukaryotic chassis

Host	Manipulation	CCM involved	Products	References
*S. cerevisiae*	Introduction of PHK pathway	Glycolysis and PPP	Farnesene	[23]
*S. cerevisiae*	Introduction of PHK pathway	Glycolysis and PPP	Polyhydroxybutyrate	[25]
*S. cerevisiae*	Introduction of PHK pathway	Glycolysis and PPP	p-Hydroxycinnamic acid	[30]
*S. cerevisiae*	Introduction of PHK pathway and A-ALD	Glycolysis and PPP	β-Amyrin	[38]
*S. cerevisiae*	Introduction of PHK pathway, overexpression of ADH2, ALD6 and acs_SE_^L641P^	Glycolysis and PPP	Fatty acid ethyl esters	[29]
*S. cerevisiae*	Introduction of PHK pathway, multi-copy integration of Tal1 and Tkl1	Glycolysis and PPP	Protopanaxadiol	[33]
*S. cerevisiae*	Introduction of PK pathway	Glycolysis and PPP	Tyrosol and salidroside	[31]
*S. cerevisiae*	Overexpression of a modified PDH pathway	Glycolysis	Acetyl-CoA	[36]
*S. cerevisiae*	*PDC1* deletion	Glycolysis	Pyruvate and lactate	[39]
*S. cerevisiae*	*PDC1* deletion	Glycolysis	2,3-Butanediol	[40]
*S. cerevisiae*	Deletion of *HXK2* and *GLK1*, tTA-controlled expression of *HXK1*	Glycolysis	Gluconate	[41]
*S. cerevisiae*	Introduction of PHK pathway, downregulation of phosphoglucose isomerase, and overexpression Acc1 and Mcr	PPP	3-Hydroxypropionic acid	[34]
*S. cerevisiae*	Repression of Ric1 and overexpression of Rki1	PPP	Shikimic acid, muconic acid and protocatechuic acid	[46]
*S. cerevisiae*	Introduction of ACL from *A. nidulans*	TCA cycle	Mevalonate	[35]
*S. cerevisiae*	Knockout of 15 genes of CCM	Glycolysis, PPP and TCA cycle	Triacetic acid lactone	[43]
*P. pastoris*	Overexpression of Zwf, Pgi and Pfk1	Glycolysis	Inositol	[42]
*P. pastoris*	Control CCM by a synthetic constitutive transcriptional signal amplification device	Glycolysis	Monacolin J	[44]
*P. pastoris*	Introduction of PHK pathway and the ACL from *M. musculus*	Glycolysis and TCA cycle	Free fatty acids and fatty alcohol	[28]
*Y. lipolytica*	Introduction of PHK pathway and PFK deletion	Glycolysis	Lipid	[26]
*C. synechocystis*	Expression of extra copies of PEPC	TCA cycle	Ethylene	[47]
*C. synechocystis*	Expression of extra copies of PEPC and elevated culture temperature	Glycolysis and TCA cycle	Succinate	[48]

### Optimization of CCM in other eukaryotic chassis

In cyanobacterium *Synechocystis* sp., the lower carbon flux of the TCA cycle limits the biosynthesis of target products. Increased copy number of the phosphoenolpyruvate carboxylase (PEPC) gene and increased carbon flux into the TCA cycle significantly increased the production of ethylene to 10.5 µg/mL/OD/day), a 1.64-fold increase compared to the original strain [47]. *Synechocystis* sp. is also an excellent chassis strain for succinate production. Overexpression of PEPC increased succinate yields to 162.3 mg/L after increasing temperature to decrease glycolytic carbon flux and increase TCA cycle carbon flux [48].

### Application of CCM optimization in ***E. coli***

#### Rearrangement of CCM metabolic flux through the PTS system

When *E. coli* uses glucose as a carbon source, the sugar phosphotransferase system (PTS) transfers the phosphate group from phosphoenolpyruvate (PEP) to glucose to produce G6P and pyruvate. PTS is the primary PEP consumption pathway for *E. coli* growth [49]. PEP is a key intermediate that links the three major CCM pathways and regulates the expression of some CCM genes [50]. Therefore, regulation of CCM by the PTS system (primarily by deletion of the PTS system) is a widely used CCM regulation strategy in *E. coli* metabolic engineering.

The deletion of the PTS system in *E. coli* decreased its carbon consumption, and inhibiting alanine: H^+^ symporter activity attenuated cell growth inhibition. These optimizations increased β-alanine production to 4.36 g/L [51]. The deletion of the PTS system and knockdown of the transcription factor TyrR, which has a repressive effect on the aromatic amino acid synthesis pathway, significantly increased L-tyrosine production [52, 53]. Further metabolic engineering optimization resulted in melanin and L-dopa yields of 3.22 g/L and 25.53 g/L, respectively [53, 54]. The knockdown of pyruvate kinase (PYK), PEPC, and malic enzymes along with deletion of the PTS system can increase the metabolic flux of the TCA cycle, resulting in a final yield of 5.89 mmol g/DCW (dry cell weight)/h of succinate in the optimized strain [55]. The replacement of the PTS system in *E. coli* with the galactose transport system resulted in a substantial accumulation of PEP. Further use of PEPC to reroute the metabolic flux of glycolysis to the TCA cycle resulted in a fumaric acid yield of 1.53 g/g dry cell weight [56]. Displacement of the PTS system of *E. coli* and overexpression of PK increased the precursors of aromatic amino acid synthesis pathway, PEP and E4P. The resulting engineered strain produced 41.7 g/L of tryptophan after fermentation in a 5 L bioreactor [57].

### Optimization of CCM by key enzymes or regulatory factors in ***E. coli***

#### Optimization of key enzymes

In *E. coli*, PGI and ZWF regulate the metabolic flux into the glycolytic pathway and PPP, respectively. Therefore, CCM optimization by regulation of PGI and ZWF is common in *E. coli* metabolic engineering. Simultaneous knockdown of PGI and ZWF can increase metabolic flux to the methylerythritol 4-phosphate (MEP) pathway, resulting in the accumulation of isoprenoids and their derivatives and facilitating terpenoid biosynthesis. In high-lycopene production strains constructed using this strategy, lycopene yields of 6.85–7.55 mg/g DCW were achieved [58, 59]. PGI knockdown can increase the metabolic flux to the PPP. Furthermore, ACS overexpression and other optimization methods increased the production of riboflavin to 585.2 mg/L [60]. ZWF knockdown increased the metabolic flux to glycolysis, and further optimization resulted in β-carotene production of 266.4 mg/L [61]. Triosephosphate isomerase (TPIA) is an enzyme that converts dihydroxyacetone phosphate (DHAP) to glyceraldehyde-3-phosphate (GAP). TPIA and ZWF knockdown shift metabolic flux toward pyruvate synthesis, and further optimization can increase 3-HP production by 4.4-fold [62].

CRISPR/Cas9 technology for large-scale gene editing of CCM pathways was used for the optimization of CCM. The use of CRISPR/Cas9 technology to silence a dozen enzymes in the glycolytic and TCA cycle pathways could redirect metabolic flux to the malonyl-CoA synthesis pathway that subsequently increases (2 S)-naringenin production to 421.6 mg/L, a 7.4-fold increase compared to the control strain [63]. Based on CRISPR silencing technology, high-throughput screening of enzymes in the CCM and related pathways that affect the yield of the target product and editing of all genes encoding these enzymes can significantly increase the yield of the target product. This CCM optimiz is currently used in the biosynthesis of (2 S)-pinocembrin and medium-chain fatty acids [64, 65].

**Fig. 2:**
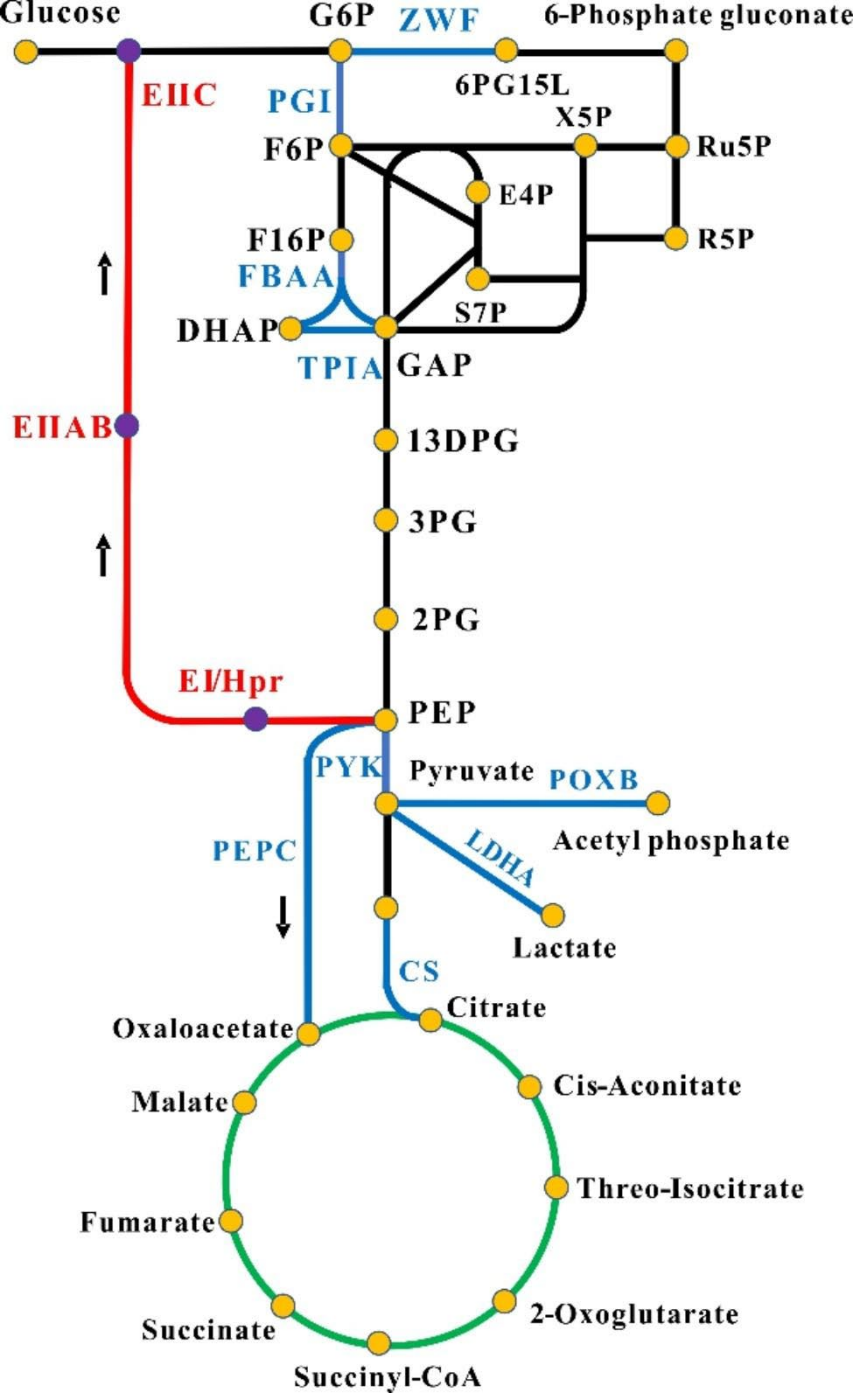
The schematic diagram of the CCM with modulated enzymes in *E. coli*. The black line represented glycolysis and pentose phosphate pathway, and the green line represented the tricarboxylic acid cycle. The red line represented the glucose PTS system and the blue line represented the pathway catalyzed by modulated enzymes. The orange dot represented the products in CCM, while the purple dot represented the products in glucose PTS system. G6P, Glucose 6-phosphate; F6P, Fructose-6-phosphate; F1,6P, Fructose-1,6-bisphosphate; GAP, Glyceraldehyde 3-phosphate; DHAP, Dihydroxyacetone phosphate; 13DPG, 3-Phospho-D-glyceroyl phosphate; 3PG, 3-Phosphoglycerate; 2PG, 2-Phosphoglycerate; PEP, Phosphoenolpyruvate; 6PG15L, 6-Phospho D-glucono-1,5-lactone; X5P, Xylulose 5-phophate; Ru5P, Ribulose 5-phophate; R5P, Ribose 5-phosphate; E4P, Erythrose 4-phosphate; S7P, Sedoheptulose 7-phophate; EI, Phosphotransferases enzyme I; Hpr, Histidine phosphate carrier protein; EIIAB, Phosphotransferases enzyme II A and B; EIIC, Phosphotransferases enzyme II C; PGI, Glucose-6-phosphate isomerase; ZWF, Glucose-6-phosphate dehydrogenase; PYK, Pyruvate kinase; PEPC, Phosphoenolpyruvate carboxylase; TPIA, Triosephosphate isomerase; FBAA, Fructose-bisphosphate aldolase; CS, Citrate synthase; LDHA, Lactate dehydrogenase; POXB, Pyruvate oxidase

CCM can also be optimized by regulating the synthesis of key CCM intermediates such as acetyl-CoA and pyruvate. Acetyl-CoA enters the TCA cycle via citrate generation, a process that depletes acetyl-CoA. Overexpression of TPIA and fructose-bisphosphate aldolase (FBAA) decreases acetyl-CoA flux to the TCA cycle, resulting in a 3-fold increase in PHB concentration in *E. coli* [66]. In addition, the introduction of an efficient citrate synthase (CS) mutant could also reduce the consumption of acetyl-CoA by the TCA cycle, shifting the metabolic flux to acetate synthesis, which yields 0.24 g/L on glucose in *E. coli* [67]. Similarly, carbon flux from acetyl-CoA can be transferred to pyruvate by creating the pyruvate dehydrogenase complex mutant. This mutant was overexpressed in *E. coli*, and by knocking down lactate dehydrogenase (LDHA) and pyruvate oxidase (POXB), it was able to accumulate 17.1 g/L of pyruvate in fermentation [68]. A summary of the applications of CCM with modulated enzymes in *E. coli* was provided in Fig. 2.

#### Optimization of key regulatory factors

The overexpression of the *D. radiodurans* response regulator DR1558 in *E. coli* increased its tolerance to oxidative stress. Moreover, the upregulated expression level of genes involved in CCM and increased accumulation of NADPH form PPP induced by this regulator resulted in PHB production of 5.31 g/L [24].

#### Other optimization methods

CCM can also be regulated by the switch system. A temperature-sensitive switch system can divide the *E. coli* fermentation process into growth and production phases. The system regulates the expression of pyruvate carboxylase and oxaloacetic decarboxylase at different temperatures, rebalancing the carbon flux between pyruvate and oxaloacetate. The introduction of this switch system resulted in a record L-threonine yield of 124.03% [70]. Another metabolic switch inhibits the metabolic flux from glycolysis to the TCA cycle and promotes a significant accumulation of acetyl-CoA, yielding 54.2 ± 1.5 mM of the target compound 3-HP with productivity of 32.1 ± 1.3% [71].

### CCM optimization in ***Corynebacterium glutamicum***

*C. glutamicum* is a chassis-engineered strain used for the biosynthesis of polyphenols, where an insufficient supply of acetyl-CoA is an important limiting factor for increasing polyphenol product yield. A moderate reduction of carbon flux in the TCA cycle can effectively decrease the consumption of acetyl-CoA. A 90% reduction in the catalytic activity of CS in the TCA cycle could lead to a 10-fold increase the yield of the target compound naringenin to 19 mg/L [72].

PEP is a key precursor in various biosynthetic pathways. Therefore, reducing PEP depletion can significantly increase the yield of related target products. The knockdown of PEPC in *C. glutamicum* resulted in a 9.3% increase in the (3R)-acetoin yield to 11.96 g/L [73]. The same strategy elevated isopropanol yield by 1.42-fold [74]. Another common method for decreasing carbon flux consumption by PEP is to optimize the PTS transport system. Knocking out the PTS system and introducing inositol transporter proteins lacking the IolR regulators restored glucose uptake and increased L-serine production to 26.40 g/L [75]. The introduction of mutant inositol transporter proteins in the retained PTS system did not increase hydroxybenzoic acid (HBA) production, but it did shorten the incubation time to achieve the maximum yield. Further suppression of CS expression significantly increased the production rate and yield of hydroxybenzoic acids, reaching 3.1 g/L within 48 h [76].

### CCM optimization in ***Bacillus licheniformis***

Because of its robustness and rapid growth, *B. licheniformis* is widely used as a chassis strain for the synthesis of various biochemicals. Optimization of *B. licheniformis* CCM can effectively increase the yield of poly-γ-glutamic acid (γ-PGA). Overexpression of PDH and CS directed more carbon fluxes to the TCA cycle, resulting in γ-PGA yields of 34.93% and 11.14%, respectively. Furthermore, knockdown of pyruvate formate-lyase reduced bypass depletion and increased γ-PGA yield by 30.70% [77]. In 2-PE biosynthesis, knockdown of PYK significantly increased PEP supply capacity and allowed for a 79% increase in 2-PE production (0.50 g/L). Deleting the PTS system and replacing it with a transporter system that does not consume PEP could increase the yield of 2-PE [78].

### CCM optimization in other prokaryotic chassis

*Pseudomonas putida* has a substantial advantage in the production of some toxic products due to its high tolerance to organic solvents. The introduction of a promoter-optimized rhamnolipid synthesis pathway in *P. putida* could direct the metabolic flux of glycolysis and acetyl-CoA to rhamnolipid synthesis, leading to a one-fold increase in rhamnolipid production to 3 g/L [79]. Regulation of CS and ACC gene expression in *P. putida* using CRISPR/Cas9 technology resulted in an 8-fold increase in acetyl-CoA production [80]. The development of pyruvate-responsive genetic pathways in *B. subtilis* enables autonomous dynamic control of CCM, resulting in 527 mg/L glucaric acid production, a 154% increase compared to the control strain [81]. The knockdown of the glucose-6-phosphate dehydrogenase gene in *Actinosynnema pretiosum* reduced the carbon flux in PPP and resulted in a 3-fold increase in the production of ansamitocins [82]. The use of PDC in the development of a CCM metabolic flux control valve device in *Zymomonas mobilis* resulted in the efficient synthesis of lactate and isobutanol, with lactate and isobutanol production reaching 70% and 65% of the theoretical maximum, respectively [83]. The knockdown of the fructose PTS system in *Mannheimia succiniciproducens* attenuates the inhibition of carbon catabolism and increases the availability of pyruvic acid. Ultimately, succinate production could be increased to 69.2 g/L by reconstituting the succinate biosynthetic pathway [84].

After knocking out the transcriptional regulator of glyceraldehyde-3-phosphate dehydrogenase and phosphoenolpyruvate carboxykinase CcpN_Gtg_ in *Geobacillus thermoglucosidasius*, the CCM metabolic flux was redirected to the riboflavin biosynthetic pathway, resulting in a 1.51-fold increase in riboflavin production from 171.6 mg/L to 260.3 mg/L [85]. Overexpression of Sp13016, a transcriptional regulator of the glycolytic pathway in *Saccharopolyspora pogona*, increased the metabolic flux in glycolysis, and decreased the metabolic flux in the TCA cycle and PPP, and resulted in the accumulation of pyruvate and acetyl-CoA, and subsequently increased butenyl-spinosyn production [86]. The development of sensor-assisted transcriptional regulation engineering utilizing the transcriptional regulator QscR in *Methylobacterium extorquens* increased acetyl-CoA production by approximately 7%, with mevalonate production reaching 2.67 g/L [87]. The applications of CCM optimization in prokaryotic chassis were listed in Table 2.

**Table 2:** Optimization of CCM in prokaryotic chassis

Host	Manipulation	CCM involved	Products	References
*E. coli*	Deletion of PTS system and TyrR repressor	Glycolysis	L-Tyrosine	[52]
*E. coli*	Deletion of PTS system and TyrR repressor	Glycolysis	Melanin	[53]
*E. coli*	Deletion of PTS system and TyrR repressor	Glycolysis	L-DOPA	[54]
*E. coli*	Deletion of PTS system and repression of the activity of Alanine: H^+^ symporter	Glycolysis	β-alanine	[51]
*E. coli*	Deletion of ZWF and TPIA	Glycolysis	3-Hydroxypropionic acid	[62]
*E. coli*	Construction of variants of the pyruvate dehydrogenase complex and deletion of LDHA and POXB	Glycolysis	Pyruvate	[68]
*E. coli*	Regulation of carbon distribution by a thermal switch system	Glycolysis	L-Threonine	[70]
*E. coli*	Design and construction of non-oxidative glycolysis pathway	Glycolysis	Acetyl-CoA	[69]
*E. coli*	Silence of a dozen or more CCM enzymes by CRISPR system	Glycolysis and TCA cycle	(2 S)-Naringenin	[63]
*E. coli*	High-throughput screening of CCM key enzymes and fine-tuning of coding genes by CRISPR silencing system	Glycolysis and TCA cycle	(2 S)-Pinocembrin	[64]
*E. coli*	High-throughput screening of CCM key enzymes and fine-tuning of coding genes by CRISPR silencing system	Glycolysis and TCA cycle	Medium chain fatty acids	[65]
*E. coli*	Expression of response regulator DR1558 from *D. radiodurans*	Glycolysis and TCA cycle	Poly‑3‑hydroxybutyrate	[24]
*E. coli*	Introduction of metabolic toggle switch	Glycolysis and TCA cycle	3-Hydroxypropionic acid	[71]
*E. coli*	Replacement of PTS system with galactose translocation system	Glycolysis and TCA cycle	Fumaric acid	[56]
*E. coli*	Substitution of PTS system with independent glucose transport system and overexpression of PK	Glycolysis and PPP	L-tryptophan	[57]
*E. coli*	ZWF deletion	Glycolysis and PPP	Lycopene	[59]
*E. coli*	ZWF deletion	Glycolysis and PPP	β-Carotene	[61]
*E. coli*	Overexpression of TPIA and FBAA	TCA cycle	Poly‑3‑hydroxybutyrate	[66]
*E. coli*	Introduction of the efficient citrate synthase variant	TCA cycle	Acetate	[67]
*E. coli*	Deletion of PTS system, PYK, PEPC and Malic enzyme	TCA cycle	Succinate	[55]
*E. coli*	Deletion of PGI and overexpression of ACS	PPP	Riboflavin	[60]
*C. glutamicum*	PEPC deletion	Glycolysis	(3R)-Acetoin	[73]
*C. glutamicum*	PEPC deletion	Glycolysis	Isopropanol	[74]
*C. glutamicum*	Deletion of PTS system and depression of myo-inositol catabolism repressor IolR	Glycolysis	L-Serine	[75]
*C. glutamicum*	Introduction of myo-inositol/proton symporter variant and downregulate of Cs	Glycolysis	Hydroxybenzoic acids	[76]
*C. glutamicum*	Reduction of CS catalysis activity	TCA cycle	Naringenin	[72]
*B. licheniformis*	Deletion of PTS system and PYK	Glycolysis	2-phenylethanol	[78]
*B. licheniformis*	Overexpression of PDH and Cs and deletion of pyruvate formate-lyase gene	TCA cycle	Poly-γ-glutamic acid	[77]
*Z. mobilis*	Ectopic expression of PDC to construct a CCM control-valve	Glycolysis	Lactate and isobutanol	[83]
*M. succiniciproducens*	Deletion of fructose PTS system	Glycolysis	Succinic acid	[84]
*G. thermoglucosidasius*	Deletion of transcriptional regulator ccpN_Gtg_	Glycolysis and PPP	Riboflavin	[85]
*B. subtilis*	Establishment of a pyruvate-responsive genetic circuit	Glycolysis and TCA cycle	Glucaric acid	[81]
*P. putida*	Introduction of genes for rhamnolipid synthesis	Glycolysis and TCA cycle	Rhamnolipid	[79]
*S. pogona*	Overexpression of the transcriptional regulator Sp13016	Glycolysis, PPP and TCA cycle	Butenyl-spinosyn	[86]
*A. pretiosum*	ZWF deletion	PPP	Ansamitocins	[82]
*M. extorquens*	Construction of a sensor by transcriptional regulator QscR	PPP and TCA cycle	Acetyl-CoA and Mevalonate	[87]

## Conclusions

The CCM-based optimization strategy can rearrange the metabolic flux in various microbial host strains to increase the supply of precursors in the biosynthetic pathway of target compounds, thereby improving the substrate conversion rate. Therefore, it is critical to maximize the production potential of microbial cell factories and improve compound production efficiency. This strategy is currently being employed to increase the yield of target compounds through metabolic engineering optimization in various chassis strains. Notably, in eukaryotic hosts, optimization of all the three CCM pathways could be used for the biosynthesis of acetyl-CoA derived compounds (e.g., terpenoids and fatty acids derivatives). In the meantime, the optimization of glycolysis and TCA cycles can also be available for the production of organic acids (e.g., pyruvate and succinate), and PPP optimization is used for the metabolic engineering of shikimic acid and aromatic amino acid derivatives (e.g., tyrosol and p-hydroxycinnamic acid). While in prokaryotic hosts, manipulation of glycolysis and PPP is used for the production of a wide range of compounds, including different amino acids (e.g., L-tyrosine and L-threonine) and acetyl-CoA derived chemicals (e.g., terpenes). And the optimization TCA cycle is available for the biosynthesis of organic acids and flavonoids (e.g., naringenin).

However, the regulatory mechanism of CCM is not yet clear due to a large number of genes included, resulting in insufficient CCM optimization approaches. CCM optimization strategies at this stage typically focus on a few functionally defined and repeatedly validated methods, limiting the wide application of CCM optimization in metabolic engineering. These issues can be addressed in the future in the following ways:

1. To deepen our understanding of CCM regulation network based on species and genetic diversity. As the regulation of CCM is sophisticated, the exploration of regulation mechanisms of CCM from the microbial hosts and other species will be helpful in developing more manipulation methods for CCM optimization.
2. To develop more transcriptional regulatory factors and gene expression regulatory switches. These regulatory elements can be used to optimize multiple genes and promote the bottleneck reaction(s) of rate-limiting enzyme(s) involved in CCM.
3. To reduce or eliminate precursor/product consuming pathways related to CCM by balancing metabolic flux or regulating metabolic pathway genes dynamically using key enzymes at metabolic pathway intersections.

## Data Availability

Not applicable.
